# Molecular Detection of Virulence-Associated Markers in *Campylobacter coli* and *Campylobacter jejuni* Isolates From Water, Cattle, and Chicken Faecal Samples From Kajiado County, Kenya

**DOI:** 10.1155/2024/4631351

**Published:** 2024-08-13

**Authors:** Daniel W. Wanja, Christine M. Mbindyo, Paul G. Mbuthia, Lilly C. Bebora, Gabriel O. Aboge

**Affiliations:** ^1^ Department of Veterinary Pathology Microbiology and Parasitology Faculty of Veterinary Medicine University of Nairobi, P.O. Box 29053-00625, Kangemi, Nairobi, Kenya; ^2^ Department of Veterinary Pathology Microbiology and Parasitology Faculty of Veterinary Medicine and Surgery Egerton University, P.O. Box 536-20115, Egerton, Kenya; ^3^ Department of Public Health Pharmacology and Toxicology Faculty of Veterinary Medicine University of Nairobi, P.O. Box 29053-00625, Kangemi, Nairobi, Kenya

**Keywords:** *Campylobacter*, campylobacteriosis, foodborne, livestock, multiple virulence genes, public health, zoonotic

## Abstract

*Campylobacter* is a zoonotic foodborne pathogen that is often linked with gastroenteritis and other extraintestinal infections in humans. This study is aimed at determining the genetic determinants of virulence-encoding genes responsible for flagellin motility protein A (*flaA*), *Campylobacter* adhesion to fibronectin F (*cadF*), *Campylobacter* invasion antigen B (*ciaB*) and cytolethal distending toxin (cdt) A (*cdtA*) in *Campylobacter* species. A total of 29 *Campylobacter coli* isolates (16 from cattle, 9 from chicken, and 4 from water samples) and 74 *Campylobacter jejuni* isolates (38 from cattle, 30 from chicken, and 6 from water samples) described in an earlier study in Kajiado County, Kenya, were examined for the occurrence of virulence-associated genes using polymerase chain reaction (PCR) and amplicon sequencing. The correlations among virulence genes were analyzed using Pearson's correlation coefficient (*R*) method. Among the 103 *Campylobacter* strains screened, 89 were found to harbour a single or multiple virulence gene(s), giving an overall prevalence of 86.4%. *C. jejuni* strains had the highest prevalence of multivirulence at 64.9% (48/74), compared to *C*. *coli* (58.6%, 17/29). The *ciaB* and *flaA* genes were the most common virulence genes detected in *C*. *jejuni* (81.1% [60/74] and 62.2% [46/74], respectively) and in *C. coli* (each at 62.1%; 18/29). *Campylobacter* isolates from chicken harboured the most virulence-encoding genes. *C. jejuni* strains from chicken and cattle harboured the highest proportions of the *cdtA* and *ciaB* genes, respectively. All the *C. coli* strains from water samples harboured the *cadF* and *flaA* genes. The results obtained further revealed a significant positive correlation between *cadF* and *flaA* (*R* = 0.733). *C. jejuni* and *C. coli* strains from cattle, chicken, and water harbour virulence markers responsible for motility/colonization, invasion, adherence, and toxin production, evoking their important role in campylobacteriosis development among humans and livestock. The identification of cattle, chicken, and water samples as reservoirs of virulent *Campylobacter* spp. highlights the possible risk to human health. These data on some virulence genes of *Campylobacter* will assist food safety and public health officials in formulating policy statements.

## 1. Introduction

*Campylobacter* spp. particularly *Campylobacter coli* and *Campylobacter jejuni* are major causative agents of bacterial gastroenteritis infections among humans in developing and developed countries [1]. Livestock including chicken play a significant role in *Campylobacter* infections in humans. The role of cattle and chicken as sources of *Campylobacter* infections in humans is related to (1) contaminated foods such as raw milk, beef, and poultry meat; (2) environmental and water pollution; and (3) direct spread to humans from infected animals. Of these, uncooked or undercooked chicken meat is the most likely source of *Campylobacter* spp. [2], often linked with sudden and unpredictable cases of human campylobacteriosis. There are also bovine-related epidemics where unpasteurized milk and its byproducts are the second most prevalent sources of infection [3]. However, other reservoirs of infection, such as companion animals, raw milk, and contaminated water, have been noted [4]. Direct contamination of milk might occur via either faeces or as a result of udder infection caused by these pathogens [5, 6].

*Campylobacter* spp. not only impact human health but also cause significant infections in livestock. *Campylobacter* infections in humans and livestock present a variable degree of virulence: ranging from asymptomatic carriage in poultry to gastroenteritis and/or watery diarrhea and sometimes extraintestinal infections (respiratory failure and severe neurological dysfunction) in humans, to mastitis, enteritis, and abortion in cattle [7]. However, the precise role of *Campylobacter* isolates in the causation of all these clinical syndromes is unclear, and additional research is consequently necessary [8]. The pathogenesis of campylobacteriosis is multifaceted and still not well understood [9]. Exploring the molecular basis of the virulence markers linked with the pathogenicity of *Campylobacter* spp. is essential for controlling diseases and/or clinical manifestations caused by this bacterium [10]. However, polymerase chain reaction (PCR) virulotyping of *Campylobacter* spp. has been widely investigated in other countries [11]. Consequently, some studies have explored the potential virulence and/or survival factors necessary for the pathogenicity of *Campylobacter* spp. [12]; the response to stress, flagellum-facilitated motility, adhesion and binding, invasion and adherence to epithelial cells of the intestines, chemotaxis, ability to produce toxins, and ability to overcome host defense cells are now known to be involved in pathological processes and/or disease development in *Campylobacter* spp. [11–13]. However, it is not clear whether specific disease syndromes correlate with a particular virulence-encoding gene. Nevertheless, virulence attributes are believed to contribute to the organism's pathogenicity and provide the ability to adhere to receptor cells on the host during disease pathogenesis, hence helping to modulate the clinical manifestations of the disease [14].

A number of genes and gene products have been documented to play vital roles during disease development. *Campylobacter* invasion antigen B (*ciaB*) is an important heat shock protein-encoding gene [15] that is necessary for invasion of the epithelium and subsequent establishment in avian intestines [16]. However, effective invasion and establishment of *Campylobacter* spp. into host epithelial cells depend on its adhesion to fibronectin F, which is encrypted by the *Campylobacter* adhesion to fibronectin F (*cadF*) gene and is responsible for the binding of this bacterium to the intercellular matrix of epithelial cells in the intestines during the disease development process [13]. Cytolethal distending toxins (cdts) are enciphered by a family of genes, namely, cdt A (*cdtA*), *cdtB*, and *cdtC*, and are essential for the cytotoxicity and destruction of intestinal absorptive cells in the host [17]. The flagellum motility protein is encrypted by the flagellin motility protein A (*flaA*) gene and is implicated in motility, establishment, autoagglutination, and biofilm development, thus contributing to the *Campylobacter* infection process in a susceptible host [18].

*Campylobacter* strains originating from different sources, including livestock and their environment, may be transferred to humans, causing gastroenteritis, among other clinical syndromes. Therefore, it is imperative to establish whether *Campylobacter* strains recovered from these sources possess virulence properties. Furthermore, virutyping *Campylobacter* isolates could be an important step in understanding the progression of associated infections in humans. Therefore, surveillance of virulence genetic determinants in *Campylobacter* spp. is exceedingly applicable to consumer health. In anticipation of this context, the objective of the present study was to establish the occurrence of virulence-encoding genes responsible for *flaA*, *cadF*, *ciaB*, and *cdtA* in *Campylobacter* species recovered from cattle, chicken, and water samples from Kajiado County, Kenya.

## 2. Materials and Methods

### 2.1. Ethical Approval

The study was reviewed and authorized by the Biosafety, Animal Use and Ethics Committee, Faculty of Veterinary Medicine, University of Nairobi (FVM/BAUEC/2020/274).

### 2.2. Study Area, Design, Source, and Culture Conditions of *Campylobacter* Strains

**Figure 1:**
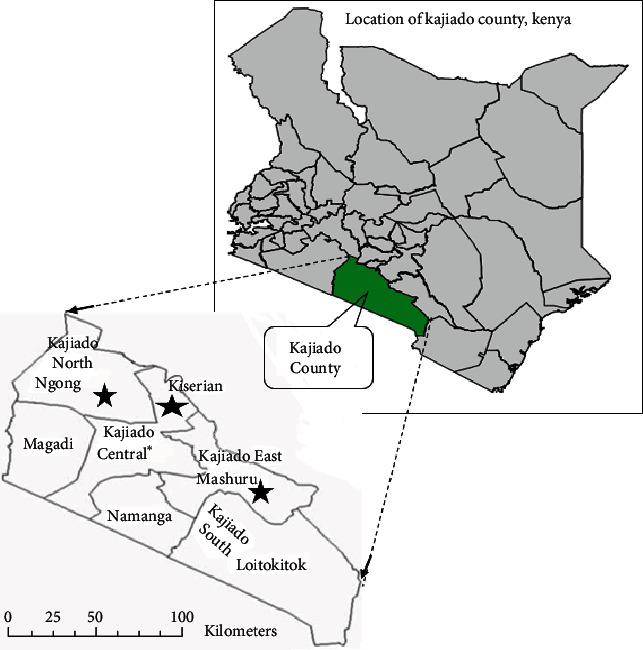
A map showing the location of Kajiado County, Kenya (black stars show study locations).

Bacterial strains utilized in the virulotyping assays were obtained from a cross-sectional investigation on seasonal occurrence on thermotolerant *Campylobacter* spp. isolated from cattle rectal swabs, chicken cloacal swabs, and water samples from cattle drinkers and other designated cattle's waterpoints, conducted in Kajiado County, southwestern Kenya. The study site locations, procedure of sampling, isolation, and identification of the isolates were as described previously [19, 20]. Briefly, a prevalence survey of 55 mixed livestock farms (primarily keeping cattle and chicken) was carried out in three subcounties in Kajiado County (Ongata Rongai and Ngong in Kajiado North subcounty; Kiserian in Kajiado West subcounty; and Kitengela, Isinya, and Mashuru in Kajiado East subcounty) (Figure 1).

The county has well-established smallholder mixed livestock (cattle, poultry, sheep, and goats among others) production systems. The mixed farms were visited once, and a single faecal swab (from cattle and chicken) and water samples were collected either during the rainy or dry season, which spans the period between October 2020 and May 2022.

Isolation of *Campylobacter* species from water samples was via aseptic filtration through 0.45 *μ*m filter paper. Thereafter, the filter paper and faecal swabs were processed by initial selective pre-enrichment in Bolton broth (Oxoid, UK), followed by routine isolation and culture for 48 h at 42°C onto modified charcoal-cefoperazone-deoxycholate agar (mCCDA)/*Campylobacter* agar plates (Oxoid, UK) under microaerobic conditions. Pure cultures of bacteria were obtained by aseptically streaking putative colonies on freshly prepared blood agar plates with selective supplement. Putative *Campylobacter* isolates were identified via both conventional biochemical methods and singleplex-PCR. Prior to the PCR assay, culture-based confirmed *Campylobacter* isolates were subjected to DNA extraction via the boiling method and then frozen at −80°C. Singleplex-PCR identification revealed that 55.6% (90/162) were *C. jejuni* and 17.9% (29/162) were *C. coli*. A total of 103 PCR-confirmed *Campylobacter* isolates including 29 *C. coli* (16 from cattle, 9 from chicken, and 4 from water samples) and 74 *C. jejuni* (38 from cattle, 30 from chicken, and 6 from water samples) isolates were selected for the current virulence study. Subsequently, the previously cryopreserved genomic DNA from these *Campylobacter* isolates was utilized for the virutyping assays.

### 2.3. Molecular Detection of Virulence Genes

The cryopreserved genomic DNA was removed from the freezer and allowed to thaw at room temperature (24°C–26°C), prior to being subjected to virutyping assays. PCR was utilized to screen virulence-associated genes including *flaA*, *ciaB*, *cdtA*, and *cadF*. The oligonucleotide primers used were designed based on gene sequence data from previously published reports (Table 1). The PCR primers used were sourced from Inqaba Biotechnologies (Pretoria, South Africa). The primer sequences were subjected to Basic Local Alignment Search Tool (BLAST) searches against the National Center for Biotechnology Information (NCBI) database (https://www.ncbi.nlm.nih.gov) to assess their specificity.

The cryopreserved genomic DNA samples were thawed and subsequently amplified using primers specific for each of the virulence markers in a Bio-Rad thermal cycler. The final PCR mixture included 12.5 *μ*L of OneTaq® 2x PCR Master Mix (New England Biolabs), 0.2 *μ*L of each primer pair (cadF-R/F, cdtA-R/F, flaA-R/F, and ciaB-R/F), and 5 *μ*L of sample DNA. The final volume was topped up to 25 *μ*L using molecular-grade water (BioConcept®, Switzerland). The following optimized multiplex-PCR (mPCR) thermal cycling conditions for *cdtA*, *flaA*, and *ciaB* genes were used: 95°C × 5 min (initial denaturation); 30 cycles 94°C × 1 min, 57°C × 1 min (annealing); 72°*C* × 1 min; 72°C × 5 min (terminal extension). Singleplex amplification conditions for *cadF* were as described for mPCR, except that the annealing temperature was 48°C for 1 min. *C. jejuni* subsp. *jejuni* ATCC 33560 and molecular-grade water were used as positive and negative controls in each PCR run, respectively. The amplified PCR products were separated via electrophoresis on a 1.5% agarose gel, stained with ethidium bromide in 1x Tris-Borate-EDTA (TBE) buffer (Cleaver Scientific Ltd, UK), and visualized via ultraviolet light transillumination using a gel document system (GelMax® 125 UVP imager, Cambridge, UK). The sizes of the PCR amplicons were compared to that of the 100 bp DNA ladder.

### 2.4. Virulence-Encoding Amplicon Sequencing

Representative amplicons encoding virulence genes generated with each primer were purified using the QIAquick PCR Purification Kit (Qiagen) and then shipped to Inqaba Biotechnologies laboratories, Pretoria, South Africa, for sequencing using both forward and reverse primers. The raw sequences were edited, aligned, and assembled into a complementary sequence using BioEdit software. The sequences were blasted against the NCBI GenBank database for the best matches. The sequences were subsequently submitted to GenBank to obtain accession numbers.

### 2.5. Data Handling and Analysis

Data on detection of virulence-encoding gene was entered into and stored in Microsoft Excel and subsequently authenticated before descriptive and inferential statistical analyses were performed via EPI INFO software (https://www.cdc.gov/epiinfo/). Pearson's correlation coefficient (*R*) was used to establish associations among the virulence genes to determine whether the presence of any of the given genes was interconnected with the other. Chi-square and Fisher's exact tests were employed to examine whether the detected virulence markers were influenced by the source of the isolates (cattle, chickens, or water). A *p* value < 0.05 was considered to indicate statistical significance.

## 3. Results

### 3.1. Detection of Virulence Genes Among *Campylobacter* Isolates

**Figure 2:**
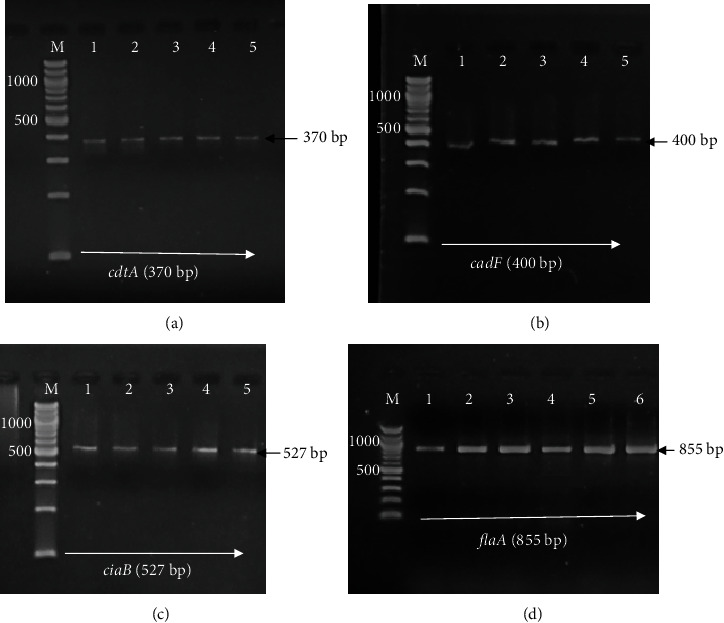
Genetic determinants of virulence factors in *Campylobacter* isolates detected by conventional PCR and 1.5% agarose gel electrophoresis: 100 bp marker (lane M). Panels (a), (b), and (c) represent isolates (1–5) positive for *cdtA* (370 bp), *cadF* (400 bp), and *ciaB* (527 bp), respectively. Panel (d) is *flaA* (855 bp).

Conventional PCR analysis revealed *Campylobacter* isolates harbouring genetic determinants responsible for various virulence factors. As shown in Figure 2, PCR bands corresponding to 370 bp for the *cdtA* gene (Figure 2(a)) and 400 bp for *cadF* (Figure 2(b)) were detected. The other virulence genes yielded specific bands corresponding to 527 bp for *ciaB* (Figure 2(c)) and 855 bp for *flaA* (Figure 2(d)).

Among the 103 *Campylobacter* strains screened, 89 (prevalence = 86.4%) were found to harbour different virulence genes: with a total of 24 strains (prevalence = 23.3%) harbouring a single virulence gene; and 65 strains (prevalence = 63.1%) harboured multiple virulence genes (two or more genes in a single sample type). *C. jejuni* strains had the highest multivirulence prevalence at 64.9% (48/74), compared to *C*. *coli* (58.6%, 17/29). *Campylobacter* strains from chicken harboured the highest prevalence of multiple virulence genes (79.5%, 31/39) than those from water (60%, 6/10) and cattle (51.9%, 28/54) (no significant difference in frequency of multivirulence genes; *p* value > 0.05).

**Figure 3:**
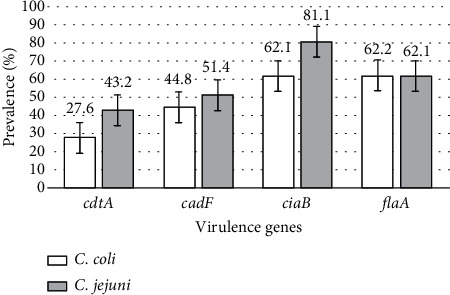
Percentages of virulence-encoding genes in *C. coli* and *C. jejuni* strains from all the sample types. The data are presented as the percentage fraction ± standard deviation (*C*. *coli* [*n* = 29], *C. jejuni* [*n* = 74]).

The frequency of detection of virulence-encoding genes among *Campylobacter* strains irrespective of the source/sample type in this study is presented in Figure 3. The *ciaB* gene was the most common virulence gene detected in *C*. *jejuni* (81.1%; 60/74) and *C. coli* (62.1%; 18/29). The frequency of *flaA* gene detection in *C. jejuni* and *C. coli* isolates was 62.2% (46/74) and 62.1% (18/29), respectively. A greater percentage of the *cadF* gene was detected in *C. jejuni* isolates (51.4%; 38/74) than in *C. coli* (44.8%; 13/29). Conversely, the *cdtA* gene was detected in 27.6% (8/29) of the *C. coli* isolates and in 43.2% (32/74) of the *C. jejuni* isolates.

### 3.2. Prevalence of Virulence-Associated Genes in *Campylobacter* Strains by Sample Type

**Figure 4:**
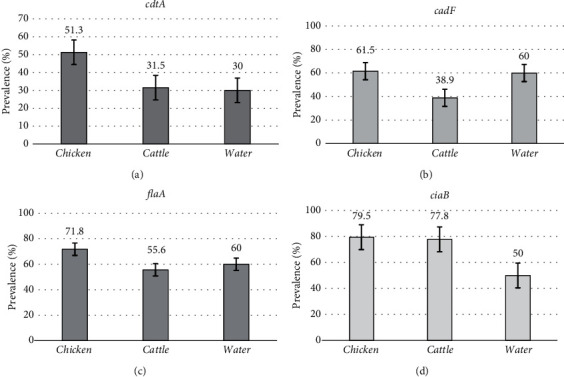
Proportion of virulence-encoding genes ((a) *cdtA*, (b) *cadF*, (c) *flaA*, and (d) *ciaB*) among *Campylobacter* isolates from cattle, chicken, and water. The data are presented as the percentage fraction ± standard deviation (chicken isolates [*n* = 51], cattle isolates [*n* = 58], and water isolates [*n* = 10]).

The proportions of *Campylobacter* strains that harboured virulence genes in different samples are shown in Figure 4. There were significant differences noted between the proportions of virulence-encoding genes found in cattle, chicken, and water (*p* value < 0.05). Chicken-*Campylobacter* strains harboured the majority of virulence-encoding genes.

### 3.3. Prevalence of Virulence-Associated Genes in *C. jejuni* and *C. coli* Isolated From Chicken, Cattle, and Water Samples

**Figure 5:**
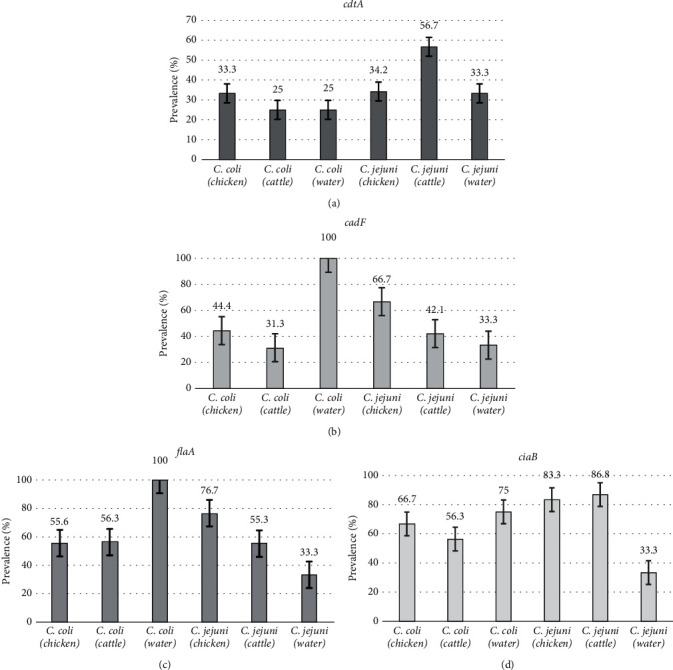
Proportion of *C. coli* and *C. jejuni* strains of cattle, chicken, and water origin harbouring the *cdtA* (a), *cadF* (b), *flaA* (c), and *ciaB* genes (d). The data are presented as the percentage proportion ± standard error*. C. coli* isolates, *n* = 29 (chicken [*n* = 9], cattle [*n* = 16], and water [*n* = 4]), and *C. jejuni* isolates, *n* = 74 (chicken [*n* = 30], cattle [*n* = 38], and water [*n* = 6]).

The results further showed variability in the proportions of virulence genes among *Campylobacter* species from the diverse sources (Figure 5). The highest detection rate of *cdtA* was detected among *C. jejuni* isolates from chicken (56.7%; 17/30), 34.2% (13/38) and 33.3% (2/6) of which were from cattle and from water, respectively. Similarly, 33.3% (3/9) of the *C*. *coli* isolates from chicken harboured *cdtA*, 25% (4/16) from cattle, and 25% (1/4) from water, as shown in Figure 5(a). All the four *C. coli* isolates from water (100%, 4/4) harboured the *cadF* gene and compared to *C. coli* isolates from chicken cloacal samples at 44.4% (4/9) and 31.3% (5/16) from cattle faecal samples, whereas *cadF* gene was detected in *C. jejuni* isolates from chicken, cattle, and water at 66.7% (20/30), 42.1% (16/38), and 33.3% (2/6), respectively, as shown in Figure 5(b). Water-derived *C. coli* and chicken-derived *C. jejuni* showed higher assemblage of *flaA* gene at 100% (4/4) and at 76.7% (23/30), respectively; as compared to *C. coli* isolates from cattle at 56.3% (9/16) and chicken at 55.6% (5/9) and *C. jejuni* strains from cattle at 55.3% (21/38) and water at 33.3% (2/6) (Figure 5(c)). A total of 33.3% (2/6), 83.3% (25/30), and 86.8% (33/38) of the *C. jejuni* isolates from water, chicken, and cattle, respectively, possessed the ciaB gene. Additionally, 75% (3/4), 66.7% (6/9), and 56.3% (9/16) of the *C. coli* isolates from water, chicken, and cattle, respectively, also harboured the *ciaB* gene (Figure 5(d)).

### 3.4. Pearson's Correlations for Virulence-Associated Genes Detected in *Campylobacter* Species

Statistically significant positive correlations were noted between several virulence genes assayed in this study (*p* < 0.05) (Table 2). The *ciaB* gene was the only gene that was not significantly correlated with the *cdtA* gene (*p* > 0.05). The presence of the *cadF* gene was strongly correlated with the presence of the *flaA* gene (*R* = 0.733). The occurrence of *cdtA* (one of the tripartite cdts), which causes unwinding of DNA strands, was moderately correlated with the presence of both the *cadF* (*R* = 0.645) and *flaA* (*R* = 0.544) genes (*p* < 0.05).

### 3.5. Nucleotide Sequence Accession Numbers

Some nucleotide sequences from this study were submitted to the GenBank and assigned accession numbers as follows: *cadF* gene (OR876350, OR876351, and OR876352) and *flaA* gene (OR876353, OR876354) in the NCBI databases available at https://www.ncbi.nlm.nih.gov/nucleotide.

## 4. Discussion

Virulence-encoding genes are responsible for *Campylobacter*'s pathogenicity; therefore, virulence-associated factors in livestock (cattle and chicken) and nonlivestock/environmental (water) reservoirs warrant studies for the sake of human safety. There are limited studies that have investigated virulence-related genes in *Campylobacter* strains of environmental origin including water, as most of them have focused on the occurrence of virulence markers in *Campylobacter* strains in humans and domestic animals, particularly poultry. This study subsequently investigated genes encoding virulence markers, including *cdtA*, *flaA*, *ciaB*, and *cadF*, in cattle, chicken, and water samples. In the current study, both *C. jejuni* and *C. coli* isolates were found to harbour multiple virulence genes at significantly higher frequencies. This finding corroborates findings in another study by Bunduruș et al. [25]. The presence of several virulence factors in a single strain increases the invasiveness of this bacterium. Consequently, the findings also imply that both *C. jejuni* and *C. coli* from the three sample types could be equally culpable for most of the clinical syndromes.

Overall, the *ciaB* gene, which is responsible for attacking host epithelial cells, was the most common virulence-encoding gene detected in this study, occurring in 79.5%, 77.8%, and 50% of the chicken, cattle, and water isolates, respectively. Thus, the chicken isolates presented the highest proportion of the *ciaB* gene. The detection rates of the *ciaB* gene in *C. jejuni* (83.3%) and *C. coli* (66.7%) isolates from chicken were comparable with the findings of previous studies [26]. However, the percentage of the *ciaB* gene among chicken isolates stated in this study was greater than that reported in other studies: 47% and 10% in *C. jejuni* and *C. coli* isolates, respectively [27], and 23.1% among *Campylobacter* isolates [28]. The *ciaB* gene was identified in 56.3% and 86.8% of the *C. coli* and *C. jejuni* strains, respectively, from cattle, which is akin to the findings of Raeisi et al. [29]. The current study further showed a high proportion (75% of *C. jejuni* and 33.3% of *C. coli*) of the *ciaB* gene in water isolates, and the results are comparable with the results of the study by Chukwu et al. [30]. On the contrary, Igwaran and Okoh [31] reported no prevalence of the same gene in *Campylobacter* isolates from water samples from both ponds/dams and rivers. The *ciaB* gene is necessary for the early phases of *Campylobacter* establishment [18]; therefore, the dominance of the latter in cattle, chicken, and water samples means that the recovered strains were able to overcome adverse intestinal conditions and instigate disease processes [18]. Additionally, this gene had a significantly low positive correlation of 19.7% and 21.2% with the *cadF* and *flaA* genes, respectively (*p* < 0.05). This finding is significant because the first stage in the pathogenesis of invasive versus toxigenic pathogens involves attachment to host cells. Thus, the *ciaB* gene is essential for pathogenic *Campylobacter* strains, which, despite lacking other virulence genes, could result in infections.

The flagellin protein FlaA, a major protein encoding the *flaA* gene, was the second most commonly detected putative virulence gene. The *flaA* gene is essential for bacterial motility and establishment in epithelial cells of the ileum. Furthermore, the *flaA* gene also accounts for *Campylobacter* attachment, attack, and establishment in host epithelial cells, thereby halting the immune response [26]. The presence of either the *flaA* or *cadF* gene results in attachment and hence increased likelihood of successful disease development. Studies have reported the presence of the *flaA* gene in *C. jejuni* and *C. coli* strains recovered from chicken [26, 32], cattle [26], and water samples [32]; the findings of this article as well conform with these studies. However, a higher incidence of up to 100% has been reported for *Campylobacter* species from diverse sources [33, 34], with higher detection of the *flaA* gene being associated with high conservation of the FlaA locus among *Campylobacter* strains.

Based on the findings of this study, 61.5%, 60%, and 38.9% of *Campylobacter* strains from chicken, water, and cattle samples, respectively, harboured the *cadF* gene. In chicken, 66.7% of *C. jejuni* and 44.4% of *C. coli* strains possessed the *cadF* gene, which is concordant with the findings of Ngobese, Zishiri, and El Zowalaty [26]. *C. coli*-*cadF* gene-possessing isolates of bovine origin were also reported by Wieczorek and Osek [33]; however, a much greater frequency (90%–100%) of the *cadF* gene in both *C. jejuni* and *C. coli* isolates has also been reported [34, 35]. All the water-derived *C. coli* isolates harboured *cadF* markers. Similarly, a greater proportion of the *cadF* gene (100%) was detected in *C. coli* strains isolated from water samples from rivers, freshwater beaches, lakes, and ponds in northern Poland [32].

*Campylobacter* spp. are known to produce cdts (encrypted by *cdtA*, *cdtB*, and *cdtC*), which cause DNA destruction, chromatin disintegration, cytoplasmic distension, and halt mitotic cell division, resulting in cumulative cellular distension and eventually pathogen-induced host necrobiosis [26]. In this study, regardless of *Campylobacter* spp., 51.3% of the *cdtA* gene was found in chicken isolates, 33.3% in *C. coli*, and 34.2% in *C. jejuni*. More or less similar findings were reported by Ramatla et al. [28], who reported that 26.9% of *C. jejuni* isolates from broilers harboured the *cdtA* gene. Ngobese, Zishiri, and El Zowalaty [26] also described a high assemblage rate of the *cdtA* gene in chicken samples, but at a much greater percentage (96% of *C. jejuni* and 83% of *C. coli*). Additionally, the *C. jejuni* isolates from cattle in the present study harboured the most *cdtA* genes (56.7%). Studies have reported discrepant detection rates—37% for *C. jejuni*, 50% for *C. coli* [26], and 100% in both *C. jejuni* and *C. coli* [33], for this gene among *Campylobacter* strains of cattle origin. Limited data exist on the occurrence of virulence markers (including *cdtA*) among *Campylobacter* strains from the environmental water. These results can subsequently be compared with findings on other cdt-encoding genes (*cdtB* or *cdtC*), where studies have detected varying incidence of the *cdtB* gene in *Campylobacter* strains found in water samples [31, 32]. The presence of cytotoxic genes (*cdtA* among others) among livestock and environmental sources highlights a food safety alarm as well as a public health warning.

Consistent with global observations, this study identified the incidence of virulence-encoding genes in *Campylobacter*, more specifically motility, CDT production, and ability to invade and adhere to epithelial cells, and these data are in accordance with studies recently conducted in developed European nations [36–40]. These studies have equally emphasized that (1) these genes mainly encipher for factors essential in the early stages of infection and (2) poultry and cattle among other animals are the primary sources of pathogenic *Campylobacter* strains to humans. The findings of the current study, however, revealed relatively greater proportions of virulence-associated genes than did other studies. This study evokes that the observed discrepancies may be due to primer specificity, PCR protocols, climate and/or environmental conditions, geographical factors and seasonality, freezing, and thawing. Additionally, these virulence-encoding genes are carried on plasmids, which may influence their occurrence in different strains [41]. Additionally, the virulence markers described in this study have also been documented in *Campylobacter* species of human origin [27, 41, 42], emphasizing the probable role of these strains in causing *Campylobacter* infections in humans. However, it is crucial to highlight that the occurrence of virulence markers is merely indicative/suggestive and unpredictable of how deadly a *Campylobacter* strain might be. To rule out this possibility, a comparative analysis of patient demographics and the interaction between virulence and presenting symptoms are needed to prove disease causation, as the severity of a disease depends on the virulence of strain involved and host immunity [43].

## 5. Conclusions

*C. jejuni* and *C. coli* strains from bovine, chicken, and water harbour multiple virulence markers responsible for motility/colonization (*flaA*), invasiveness (*ciaB*), adherence (*cadF*), and toxin production (*cdtA*), evoking their important role in campylobacteriosis development among humans and livestock. The virulence-encoding markers were more prevalent in the chicken samples than in the other sample types. Additionally, regardless of the sample type, *C. jejuni* strains exhibited the highest detection rate of multiple virulence markers in this study. Additional studies involving full-genomic sequencing to evaluate other genetic markers for virulence would assist in elaborating the possible role of chicken, cattle, and environmental waters as vehicles in the epidemiology of campylobacteriosis disease in humans.

## Figures and Tables

**Table 1:** Primers used for virulence gene typing in this study.

**Virulent gene**	**Primer**	**Primer sequence (5 **′**-3**′**)**	**Amplicon size (bp)**	**Annealing temperature**	**Reference**
*cadF*	cadF-R	R-TTG AAG GTA ATT TAG ATA TG	400	48°C	Konkel et al. [21]
	cadF-F	F-CTA ATA CCT AAA GTT GAA AC			
*cdtA*	cdtA-F	F-CCT TGT GAT GCA AGC AAT C	370	57°C	Hickey et al. [22]
	cdtA-R	R-ACA CTC CAT TTG CTT TCT G			
*flaA*	flaA-F	F-AAT AAA AAT GCT GAT AAA ACA GGT G	855	57°C	Datta, Niwa, and Itoh [23]
	flaA-R	R-TAC CGA ACC AAT GTC TGC TCT GAT T			
*ciaB*	ciaB-F	F-TGC GAG ATT TTT CGA GAA TG	527	57°C	Zheng et al. [24]
	ciaB-R	R-TGC CCG CCT TAG AAC TTA CA			

Abbreviations: F: forward; R: reverse.

**Table 2:** Assessment of Pearson's correlations for virulence-encoding genes in relation to the other genes in a single sample type in *Campylobacter* strains.

**Virulence-encoding gene**	**Statistical test**	***cdtA***	***cadF***	***ciaB***	***flaA***
*cdtA*	Pearson's correlation (*R*)	1	0.645^b^	0.172	0.540^b^
	Sig. (two-tailed)		0.000	0.082	0.000
*cadF*	Pearson's correlation (*R*)	0.645^b^	1	0.198^a^	0.733^b^
	Sig. (two-tailed)	0.000		0.045	0.000
*ciaB*	Pearson's correlation (*R*)	0.172	0.198^a^	1	0.212^a^
	Sig. (two-tailed)	0.082	0.045		0.032
*flaA*	Pearson's correlation (*R*)	0.540^b^	0.733^b^	0.212^a^	1
	Sig. (two-tailed)	0.000	0.000	0.032	

^a^Significant correlation at 0.05 level (two-tailed).

^b^Significant correlation at 0.01 level (two-tailed).

## Data Availability

The raw datasets used in this study are available from the corresponding author (wanjadanie@gmail.com) upon reasonable request.
